# Contribution of RNA/DNA Binding Protein Dysfunction in Oligodendrocytes in the Pathogenesis of the Amyotrophic Lateral Sclerosis/Frontotemporal Lobar Degeneration Spectrum Diseases

**DOI:** 10.3389/fnins.2021.724891

**Published:** 2021-09-01

**Authors:** Chiara F. Valori, Manuela Neumann

**Affiliations:** ^1^Molecular Neuropathology of Neurodegenerative Diseases, German Center for Neurodegenerative Diseases, Tübingen, Germany; ^2^Department of Neuropathology, University Hospital of Tübingen, Tübingen, Germany

**Keywords:** oligodendrocytes, ALS, FTLD, TDP-43, FUS

## Abstract

Amyotrophic lateral sclerosis (ALS) and frontotemporal lobar degeneration (FTLD) are two incurable neurodegenerative disorders, often considered as the extreme manifestations of a disease spectrum, as they share similar pathomechanisms. In support of this, pathological aggregation of the RNA/DNA binding proteins trans-activation response element DNA-binding protein 43 (TDP-43) or fused in sarcoma (FUS) is the pathological hallmark found in neurons and glial cells of subsets of patients affected by either condition (i.e., ALS/FTLD—TDP-43 or ALS/FTLD—FUS, respectively). Among glia, oligodendrocytes are the most abundant population, designated to ensheath the axons with myelin and to provide them with metabolic and trophic support. In this minireview, we recapitulate the neuropathological evidence for oligodendroglia impairment in ALS/FTLD. We then debate how TDP-43 and FUS target oligodendrocyte transcripts, thereby controlling their homeostatic abilities toward the axons. Finally, we discuss cellular and animal models aimed at investigating the functional consequences of manipulating TDP-43 and FUS in oligodendrocytes *in vivo*. Taken together, current data provide increasing evidence for an important role of TDP-43 and FUS-mediated oligodendroglia dysfunction in the pathogenesis of ALS/FTLD. Thus, targeting disrupted oligodendroglial functions may represent a new treatment approach for these conditions.

## Introduction

Amyotrophic lateral sclerosis (ALS) is the most frequent neuromuscular disease characterized by the predominant loss of first and second motor neurons in the brain and spinal cord, leading to muscle weakness, paralysis, and death generally within 1–5 years (Hardiman et al., 2017). Frontotemporal dementia (FTD) is the second most common cause of presenile dementia characterized by the predominant degeneration of the frontal and temporal lobes (i.e., frontotemporal lobar degeneration, FTLD), causing personality and behavioral changes as well as difficulties formulating and understanding language (Piguet and Kumfor, 2020). In addition to a recognized clinical overlap (Burrell et al., 2016), seminal discoveries in the last decades have provided compelling molecular evidence that ALS and FTLD are closely related conditions sharing overlapping pathogenesis with dysfunctions of RNA binding proteins emerging as key roles. Briefly, trans-activation response element DNA-binding protein 43 (TDP-43) was discovered in 2006 as a disease protein in the most common form of FTLD and in almost all ALS cases (Arai et al., 2006; Neumann et al., 2006), now renamed as FTLD—TDP and ALS—TDP. They include sporadic and genetic cases with mutations in *GRN* and *C9orf72* as the most common defects in addition to other rarer genetic forms with mutations in *TARDBP*, *TBK1*, *VCP*, *OPTN*, and UBQLN2 (Pottier et al., 2016). This was followed in 2009 by the identification of mutations in *fused in sarcoma* (*FUS*) in ∼5% of familial ALS (ALS-*FUS*) (Kwiatkowski et al., 2009; Vance et al., 2009) and the subsequent recognition of FUS aggregation together with TAF15 and EWS as hallmark lesions in ∼10% of sporadic FTLD cases (FTLD—FUS) (Munoz et al., 2009; Neumann et al., 2009b,c).

**FIGURE 1:**
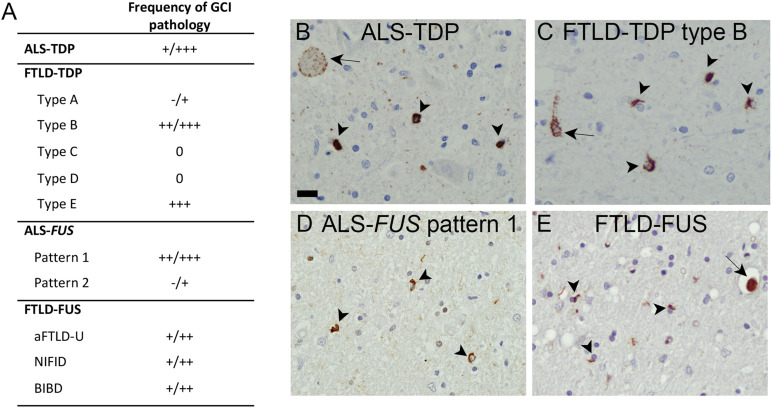
Oligodendroglial pathology in TDP-43 and FUS proteinopathies. **(A)** Glial cytoplasmic inclusions (GCI) are a characteristic feature with presence in variable amounts in affected brain regions in ALS—TDP, in subtypes of FTLD—TDP (particularly types B, E), subtypes of ALS—*FUS* (particularly in pattern 1 associated with adult onset and longer disease duration), and all distinct entities of FTLD—FUS, including aFTLD-U, basophilic inclusion body disease, and neuronal intermediate filament inclusion disease. Semiquantitative scores: 0, absent;+, rare;++, moderate; +++, abundant. **(B–E)** Immunohistochemistry of human postmortem tissues with pTDP-43 S409/410 antibody (Neumann et al., 2009a) demonstrating numerous GCI (arrowheads) in spinal cord of ALS—TDP case **(B)** and frontal cortex of FTLD—TDP type B case **(C)**, and FUS (Proteintech) immunohistochemistry demonstrating GCI in spinal cord of ALS-*FUS* case with pattern 1 pathology **(D)** and basal ganglia of FTLD—FUS (basophilic inclusion body disease) case **(E)** in addition to neuronal cytoplasmic inclusions (arrow). Scale bar in panel **B** = 20 μm.

TDP-43 and FUS the acronyms improve readability are ubiquitously expressed DNA/RNA binding proteins with a physiologically predominant nuclear localization, involved in multiple steps of RNA metabolism, including transcription, splicing, transport, and stabilization (Ratti and Buratti, 2016; Buratti, 2021). The mechanisms underlying TDP-43/FUS aggregation and neurodegeneration are complex and currently not fully understood. However, given the intimate association between cytoplasmic accumulation of abnormal TDP-43 or FUS associated with their nuclear depletion in a disease state, the current consensus is that most likely a combination of complex disturbances due to loss of nuclear and cytoplasmic functions and gain of toxic functions through aggregates is involved, with abnormal messenger RNA particle formation and dynamics as well as nucleocytoplasmic transport defects emerging as crucial factors (Bowden and Dormann, 2016; Ederle and Dormann, 2017). Although most previous and current research to dissect the pathomechanisms is focused on neurons, it has to be noted that oligodendrocytes are also strongly affected by TDP-43 and FUS pathology in ALS/FTLD (Figure 1; Neumann et al., 2007; Geser et al., 2008; Mackenzie and Neumann, 2017). Given the fact that oligodendrocytes share a developmental origin with spinal cord motor neurons (Lu et al., 2002) and are, like neurons, heavily dependent on messenger RNA (mRNA) transport over long distances for local translation, it is tempting to speculate overlapping aggregation mechanisms and that oligodendroglial dysfunction might contribute to TDP-43 and FUS disease pathogenesis as discussed for other neurodegenerative diseases (Ettle et al., 2016; Mot et al., 2018).

Here, we review the neuropathological and genetic evidence of oligodendrocyte impairment in TDP-43/FUS-proteinopathies, summarize current insights into the roles of TDP-43 and FUS in oligodendrocytes, and discuss the potential impact of altered oligodendroglial functions in ALS/FTLD pathogenesis.

## Neuropathological and Genetic Evidence for Oligodendroglial Dysfunction in Human Trans-Activation Response Element DNA-Binding Protein 43 and Fused in Sarcoma/FET Proteinopathies

Intracellular accumulation of hyperphosphorylated TDP-43 with loss of physiological nuclear TDP-43 is the hallmark in the most common form of frontotemporal lobar degeneration (FTLD—TDP) and in the vast majority of cases of ALS (ALS—TDP). Neuronal TDP-43 pathology presents as compact or diffuse neuronal cytoplasmic inclusions, neuronal intranuclear inclusions, dystrophic neurites, and thread and dot pathology. Based on the morphology, amount, and distribution pattern of cortical neuronal inclusions, at least five distinct subtypes of FTLD—TDP pathology can be delineated (types A–E), each with relatively specific clinical and genetic associations and recognition that cortical pathology in ALS—TDP most closely resembles FTLD—TDP type B (Mackenzie and Neumann, 2017; Neumann et al., 2021). The presence of additional TDP-43 pathology in oligodendrocytes as glial cytoplasmic inclusions (GCIs) (Figure 1) was already described briefly after the initial discovery of TDP-43 as disease protein in FTLD—TDP (Neumann et al., 2007) and ALS—TDP (Mackenzie et al., 2007) but was only more recently studied in more detail. In fact, GCI pathology in the gray and white matter of the spinal cord, precentral gyrus, and middle frontal gyrus is reported as a highly characteristic feature of almost all ALS—TDP cases, including sporadic and genetic forms with mutations in *C9orf72*, *TARDBP*, and *OPTN* (Brettschneider et al., 2013, 2014; Lorente Pons et al., 2020; Nolan et al., 2020). In the gray matter of the anterior horn, the amount of GCI pathology was reported to correlate with the severity of neuronal TDP-43 pathology and neuronal loss (Brettschneider et al., 2014), and in the precentral and middle frontal gyrus, the severity of GCI pathology was reported to correlate with neuronal TDP-43 pathology (Lorente Pons et al., 2020). Moreover, GCIs may be more abundant than neuronal pathology and might even precede neuronal TDP-43 pathology, suggesting that particularly gray matter oligodendroglial involvement may be an early event in the disease process (Brettschneider et al., 2014; Nolan et al., 2020). Notably, GCI pathology was mainly observed in oligodendroglia with potential close axonal contacts, whereas satellite cells located around the soma of neurons with TDP-43 pathology remain rather uninvolved (Brettschneider et al., 2013, 2014). In the context of FTLD—TDP, a recent detailed analysis on the subcortical TDP-43 pathology among FTLD—TDP subtypes demonstrated the presence of moderate to frequent GCI pathology as a highly discriminatory feature to separate FTLD—TDP type B (including sporadic and genetic cases with *C9orf72* mutations) from other FTLD—TDP types (Mackenzie and Neumann, 2020). Moreover, an association between abundant GCI pathology and shorter disease duration was reported for type E cases (Lee et al., 2017). Thus, oligodendroglial dysfunction might particularly contribute to ALS—TDP and FTLD—TDP type B and E pathogenesis. Given the increasing evidence suggesting the existence of distinct TDP-43 strains that might propagate in a prion-like manner as a molecular basis for the distinct clinical and pathological TDP-43 proteinopathies (Kawakami et al., 2019; Neumann et al., 2021), it is tempting to speculate that ALS—TDP and FTLD types B and E might share common TDP-43 strains with a specific cellular tropism for oligodendrocytes.

FUS proteinopathies include ALS cases with *FUS* mutations and three conditions presenting with FTD and/or a movement disorder, including atypical FTLD-U, neuronal intermediate filament inclusion disease, and basophilic inclusion body disease. GCI pathology is a common feature in all distinct FTLD—FUS entities (Mackenzie et al., 2011b). For ALS-*FUS*, two broad distinct neuropathological patterns based on the frequency, morphology, and distribution pattern of distinct FUS-positive inclusion types can be delineated that correlate with the clinical phenotype and severity of the functional consequence of specific *FUS* mutations (Mackenzie et al., 2011a). Notably, anatomically widespread moderate/abundant numbers of GCI with a strong correlation between the number of GCI and NCI in the affected neuroanatomical region are a highly characteristic feature for pattern 1, which is associated with longer disease duration and later disease onset (Mackenzie et al., 2011a).

Although the origin of cells with GCI in TDP-43 and FUS proteinopathies as oligodendroglia has been validated in several studies using various markers for immature and mature oligodendroglial lineage cells, including Olig2 (Nolan et al., 2020), TPPP/p25 (Rohan et al., 2014; Fatima et al., 2015), and 2′,3′-cyclic-nucleotide 3′-phosphodiesterase (Mackenzie et al., 2011a), it remains to be determined whether specific subtypes of oligodendroglial cells are more prone to develop GCI than others, an issue currently also hampered by the limited specificity/sensitivity of available antibodies in human postmortem tissues.

In addition to neuropathological findings, further evidence for a role of oligodendroglia in ALS/FTD is provided from genetic studies that have identified the oligodendrocyte-specific gene *MOBP* as a risk factor for ALS (van Rheenen et al., 2016) and a risk variant of *MOBP* to be associated with more severe white matter degeneration and a clinically more aggressive form of FTD (Irwin et al., 2014). However, this could not be verified in a subanalysis of autopsy-confirmed FTLD—TDP cases in this cohort, although the total numbers of cases might have been too small (Irwin et al., 2014). More recently, applying a polygenic risk score approach with combined analysis of genome-wide association study and single-nucleus RNA sequencing datasets uncovered the association of a distinct subpopulation of oligodendrocytes to ALS risks (Saez-Atienzar et al., 2021).

## Potential Mechanisms of Oligodendroglial Dysfunction in the ALS/FTLD Spectrum

Although oligodendrocyte biology has been extensively reviewed elsewhere (Butt et al., 2019), some key aspects relevant in the context of ALS/FTLD pathogenesis are worth mentioning here. Briefly, oligodendrocytes differentiate from oligodendrocytes precursor cells (OPCs) during embryonic and postnatal development in rodents (Takebayashi and Ikenaka, 2015) and humans (Czepiel et al., 2015). However, the central nervous system hosts an OPC pool, which constitutes 5–10% of glial cells and which retains the potential to generate new mature oligodendrocytes as well as astrocytes throughout the entire life (Fletcher et al., 2021). Recently, single-cell RNA sequencing analysis revealed that differentiation is a graded process leading to the generation of distinct subtypes of mature oligodendrocytes (Marques et al., 2016), whose most thoroughly characterized functional task is the formation and the maintenance of myelin wraps around the axons to ensure saltatory conduction. Furthermore, oligodendrocytes support the axons by providing trophic and metabolic support. Specifically, different groups identified lactate as an energy source that is transferred from oligodendrocytes to axons (Funfschilling et al., 2012; Lee et al., 2012). Finally, oligodendrocytes engage in bidirectional communications with neurons by responding to glutamatergic signaling with the neuroprotective release of extracellular vesicles such as exosomes (Fruhbeis et al., 2013, 2020; Mukherjee et al., 2020).

Because cells with TDP-43 or FUS-positive GCI displays a noticeable depletion of the physiological nuclear immunoreactivity, one can speculate that affected oligodendrocytes develop a pathological phenotype resulting from the combined action of the loss of nuclear/cytoplasmic functions and the gain of toxicity induced by the abnormal cytoplasmic expression. However, crucial questions to be resolved are as follows: What are the functional consequences, and how might oligodendroglial dysfunction contribute to degeneration? What causes/triggers TDP-43/FUS aggregation in oligodendrocytes?

**FIGURE 2:**
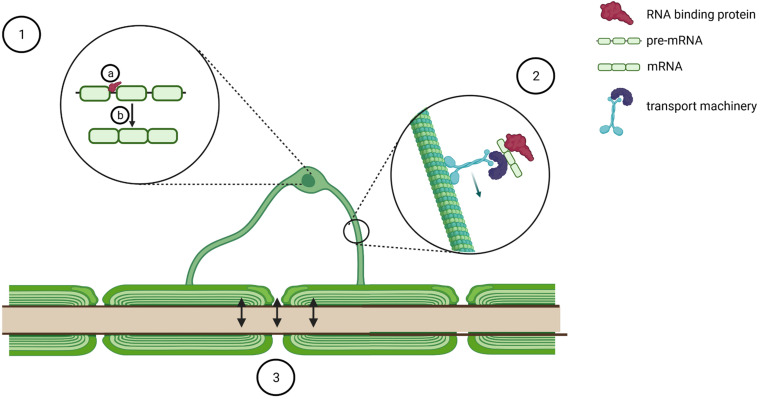
Schematic of potential mechanisms of oligodendrocyte dysfunction in pathogenesis of TDP-43/FUS proteinopathies. TDP-43 and FUS pathology in oligodendrocytes might modulate oligodendrocyte biology by binding to several transcripts (1a) and regulating pre-mRNA splicing (1b). Furthermore, it might affect transport of key RNA transcripts such as *Mbp* (2) and perturb bidirectional communication with axons (3). Created with BioRender.com.

Different aspects are discussed in the following and illustrated in Figure 2.

### Oligodendrocytes Precursor Cells Differentiation

In mice, both *Tardbp* and *Fus* are highly expressed in OPCs, and their expression decreases with oligodendrocyte maturation (Zhang et al., 2014)^1^, prompting the speculation that TDP-43/FUS might play important roles in those cells. Neural progenitor cells derived from an ALS patient with a *TARDBP* mutation differentiate *in vitro* to mature oligodendrocyte as efficiently as cells derived from non-ALS cases or other sALS and fALS patients, including those with *C9orf72* mutation, whereas mutant *FUS* carriers were not investigated (Ferraiuolo et al., 2016; Livesey et al., 2016). However, it has to be noted that these models do not recapitulate inclusion body formation with nuclear depletion of TDP-43. Unfortunately, no OPC-specific knockout models nor transgenic mice selectively expressing mutant versions of either protein have yet been generated, and therefore, we currently lack *in vivo* information as to how the loss of TDP-43/FUS might regulate OPC functions and their ability to differentiate into mature oligodendrocytes. This is of outstanding importance because the generation of new oligodendrocytes not only occurs physiologically in response to neuronal activity and it is important for learning and memory (Fletcher et al., 2021), but it is also key to restore myelin upon injury (Biname et al., 2021).

### Mature Oligodendrocytes Survival

To investigate the role of TDP-43 and FUS in mature oligodendrocytes *in vivo*, *Tardbp* (Wang et al., 2018) or *Fus* (Guzman et al., 2020) was depleted from cells expressing 2′,3′-cyclic-nucleotide 3′-phosphodiesterase. Interestingly, TDP-43 depletion leads to their demise and a compensatory hyperproliferation and differentiation of OPCs in the white matter in transgenic mice (Wang et al., 2018). In contrast, *Fus* ablation does not lead to oligodendrocyte death (Guzman et al., 2020), thus suggesting that FUS functions could be compensated by other RNA-binding proteins, whereas TDP-43 is non-dispensable in mice. These findings should be complemented by generating transgenic mice selectively expressing mutant TDP-43 or mutant FUS in mature oligodendrocytes to investigate whether mutations recapitulate the knockout phenotype or whether they induce novel toxic functions.

### Myelination

TDP-43 and FUS are likely implicated in several aspects of myelination. Firstly, cross-linking immunoprecipitation sequencing experiments discovered that TDP-43 binds to the mRNAs from key genes for myelination such as *Plp1*, *Mbp*, *Mog*, and *Mag* (Wang et al., 2018), whereas FUS binds to *MBP* (Hoell et al., 2011) and *Mal* (Lagier-Tourenne et al., 2012). Secondly, FUS depletion leads to *Mobp* alternative splicing (Lagier-Tourenne et al., 2012), whereas either TDP-43 or FUS depletion leads to alternative splicing of *Mag* exon 8 (Lagier-Tourenne et al., 2012; Kapeli et al., 2016; Figure 2), although with opposite effects, thus corroborating the hypothesis that these proteins affect oligodendrocytes with distinct modalities. Finally, although most myelin-associated transcripts are translated in the perinuclear region and then transported as mature proteins, MBP protein synthesis occurs distally, at the sites of active myelination, thus requiring the transport of *Mbp* mRNA in translationally silent RNA granules (Muller et al., 2013), membrane-less organelles arising from the liquid–liquid phase separation of intrinsically disordered RNA/DNA binding proteins such as FUS and TDP-43 (Portz et al., 2021; Figure 2). As several lines of evidence indicate that both TDP-43 and FUS are implicated in mRNA particle formation and mRNA trafficking in neurons (Thelen and Kye, 2019), one could speculate that they might contribute to the same process in oligodendrocytes. In particular, *Mbp* transport is a tightly regulated process that requires the distinct action of several RNA binding proteins, including hnRNPA2B1 (Raju et al., 2008), hnRNP K (Torvund-Jensen et al., 2014), and fragile X mental retardation protein (Doll et al., 2020). Intriguingly, TDP-43 is a well-characterized interactor of hnRNPA2 (Buratti et al., 2005; He et al., 2014; Romano et al., 2014; Chiang et al., 2020), hnRNP K (Moujalled et al., 2015, 2017), and fragile X mental retardation protein (Blokhuis et al., 2016; Majumder et al., 2016; Chu et al., 2019), which is also a FUS interacting partner (Blokhuis et al., 2016; He and Ge, 2017). In addition, indirect mechanisms could also tune *Mbp* transport. To this end, it is worth mentioning that FUS and TDP-43 regulate the alternative splicing and the stability of *Mapt* (Orozco et al., 2012; Gu et al., 2017a,b; Ishigaki et al., 2017; Wu et al., 2021), the gene coding for tau, a protein involved in the regulation of the microtubular network and necessary for *Mbp* mRNA transport as well as for the correct maturation and myelination process of oligodendrocytes (Seiberlich et al., 2015). Intriguingly, ubiquitous TDP-43^Q331K^ knock-in mice can be stratified into two populations on the basis of their behavioral impairment and affected mice display altered *Mapt* splicing pattern, whereas unaffected animals upregulate genes regulating myelination (White et al., 2018). Taken together, these data indicate oligodendrocytes as key disease modifiers and suggest that impaired focal delivery of *Mbp* could be a contributor to TDP-43 and FUS proteinopathies. In line with this, a recent paper reports that the relative protein expression of MBP *versus* PLP1 is reduced in spinal cord corticospinal tract white matter of ALS patients (Lorente Pons et al., 2020).

### Oligodendrocyte-Neuron and Oligodendrocyte-Astrocyte Interplay

In addition to myelination, oligodendrocytes provide trophic support to neurons (Duncan et al., 2021), a task that becomes defective in patient-derived oligodendrocytes. Indeed, neurons cocultured with ALS oligodendrocytes display reduced survival, even in the absence of obvious differentiation impairment (Ferraiuolo et al., 2016). Conversely, no evidence of neurotoxicity has yet been presented in mouse models where TDP-43 (Wang et al., 2018) or FUS (Guzman et al., 2020) has been depleted from oligodendrocytes. There are different possibilities to explain such discrepancy. Firstly, mutant TDP-43 might trigger oligodendrocytes dysfunction through gain of toxicity, and to date, selective expression of mutant TDP-43 or FUS in oligodendrocytes has not been investigated in transgenic mice. Alternatively, oligodendrocyte biology in animal models might not adequately recapitulate their functions in humans, an interpretation supported by single-nucleus RNA sequencing studies that revealed that only some human oligodendrocyte subpopulations have a counterpart in the rodent brain (Hodge et al., 2019). Moreover, a study comparing the transcriptome of neurons and oligodendrocytes from human and non-human primates unveiled that oligodendrocytes display a higher degree of evolution than neurons (Berto et al., 2019). Taken together, these findings urge us to interpret data gathered from mouse models with caution and underline the need for the development of human-derived models, such as brain organoids (Shaker et al., 2021), to explore both cell-autonomous and non-cell-autonomous aspects of oligodendrocyte biology.

Mechanistically, oligodendrocytes support neurons by releasing lactate (Funfschilling et al., 2012; Lee et al., 2012; Figure 2), and reduction of both oligodendrocyte and neuronal lactate transporters has been reported in the motor cortex of ALS patients (Lee et al., 2012). However, in coculture experiments, reduced lactate release from ALS/FTD oligodendrocytes only partially explains their impaired ability to support neuronal viability (Ferraiuolo et al., 2016), arguing that other mechanisms are likely contributing to the pathogenic mechanism. To this end, an interesting hypothesis arises from the trophic effect that glutamate-dependent oligodendrocyte-derived exosomes play on axons (Fruhbeis et al., 2013, 2020; Mukherjee et al., 2020) as both TDP-43 (Iguchi et al., 2016) and FUS (Kamelgarn et al., 2016) have been identified as components of exosomes purified from different cell culture supernatants. Moreover, in the plasma of sALS patients, both TDP-43 and FUS can be detected in microvesicles (Sproviero et al., 2018), and the accumulation of TDP-43 in plasma exosomes is currently under investigation as a potential progression biomarker in ALS (Chen et al., 2020). Taken together, these findings prompt several questions, which would deserve *ad hoc* investigations: which is the cellular source of TDP-43 and FUS containing exosomes? Are exosome biogenesis and release impaired? Is the composition of exosome payload perturbed in oligodendrocytes derived from ALS and FTLD patients? Finally, oligodendrocytes protect axons from the depolarizing effect of extracellular potassium accumulation by expressing the potassium channels Kir4.1 (Schirmer et al., 2018). Intriguingly, the same channels are also expressed on astrocytes, where they modulate the morphology and the electrophysiological properties of fast α-motor neurons (Kelley et al., 2018). Because ALS patient-derived astrocytes display reduced expression of Kir4.1 (Kelley et al., 2018), it would be of outstanding interest to learn whether oligodendrocytes might show the same defect.

Complementary, oligodendrocyte dysfunction in ALS/FTLD might not be exclusively cell-autonomous, but it could arise from aberrant signaling coming from other glial cell populations. In particular, the genetic ablation of *Tardbp* in astrocytes switches their transcriptome and that of the surrounding microglia toward an inflammatory status in mice (Peng et al., 2020). Furthermore, the spinal cord of those animals displays a reduced number of mature oligodendrocytes in the gray matter (Peng et al., 2020).

## Mechanisms of Selective Vulnerability of Oligodendrocytes in Amyotrophic Lateral Sclerosis/Frontotemporal Lobar Degeneration Spectrum

The neuropathological analysis showing that TDP-43 or FUS aggregates are restricted to subsets of neurons and oligodendrocytes prompts the question as to why these cell types are exquisitely vulnerable to the formation of inclusions. Although this topic is still under-investigated, one can speculate that oligodendrocyte susceptibility might arise from their high energy demand required for myelination (Tepavcevic, 2021) and active mRNA transport over long distances, exposing them to mitochondrial dysfunction and oxidative stress. In cultured oligodendrocytes, oxidative insult induces stress granules, membrane-less organelles that transiently store translationally silenced mRNA, including *Mbp* (Wang et al., 2010), and are thought to act as nucleation events for inclusion body formation in TDP-43/FUS-proteinopathies upon aberrant dynamics of assembly and disassembly (Baradaran-Heravi et al., 2020). Intriguingly, edaravone, a recently approved drug for the treatment of ALS, has been shown to protect oligodendrocytes from oxidative insults (Ueno et al., 2009; Miyamoto et al., 2013).

A complementary hypothesis might be that oligodendrocytes have a reduced proteostatic capacity because they display increased vulnerability to proteasome impairment when compared with other glial cells (Goldbaum et al., 2006). Moreover, the inactivation of the unfolded protein response leads to subsequent autophagy impairment and oligodendrocyte toxicity (Stone et al., 2020), and in the motor cortex of ALS cases, the expression of unfolded protein response target genes was found to be associated with oligodendrocytes (Montibeller et al., 2020). More specifically, TDP-43 binds to the von Hippel Lindau protein (VHL), an adapter that targets its interaction partners to the ubiquitination machinery for subsequent degradation (Uchida et al., 2016). TDP-43 depletion leads to VHL overexpression that has the paradoxical effect of promoting TDP-43 inclusion formation (Uchida et al., 2016). One could therefore speculate that an initial insult leads to stress granule formation, which in turn causes an early depletion of TDP-43 from the nucleus and upregulation of VHL. This event would hyper-stabilize TDP-43 granules converting them into pathological aggregates, which would induce further cellular stress and therefore closing a harmful vicious cycle.

## Conclusion

In summary, investigations so far have provided substantial evidence for a potential role of oligodendroglial dysfunction in ALS/FTLD. However, more detailed studies on the physiological roles of TDP-43 and FUS in the complex functions of oligodendroglial lineage cells and the determination of molecular changes during the disease process are required to further dissect the significance of specific alterations in disease pathogenesis. This might pave the way for new therapeutic approaches in ALS/FTLD by, e.g., boosting oligodendrocyte metabolism and functions.

## Author Contributions

CV and MN researched literature, conceived the presented concepts, and wrote the manuscript. Both authors contributed to the article and approved the submitted version.

## Conflict of Interest

The authors declare that the research was conducted in the absence of any commercial or financial relationships that could be construed as a potential conflict of interest.

## Publisher’s Note

All claims expressed in this article are solely those of the authors and do not necessarily represent those of their affiliated organizations, or those of the publisher, the editors and the reviewers. Any product that may be evaluated in this article, or claim that may be made by its manufacturer, is not guaranteed or endorsed by the publisher.

## Footnotes

1
https://www.brainrnaseq.org/
